# The T4 TerL Prohead Packaging Motor Does Not Drive DNA Translocation by a Proposed Dehydration Mechanism

**DOI:** 10.3390/v12050522

**Published:** 2020-05-09

**Authors:** Lindsay W. Black, Bingxue Yan, Krishanu Ray

**Affiliations:** 1 Department of Biochemistry and Molecular Biology, University of Maryland School of Medicine, Baltimore, MD 21201, USA; byan@som.umaryland.edu (B.Y.); kray@som.umaryland.edu (K.R.); 2 Institute of Human Virology, University of Maryland School of Medicine, Baltimore, MD 21201, USA

**Keywords:** T4 TerL prohead, DNA crunching, heteroduplex A-form DNA:RNA packaging, linear motor mechanism

## Abstract

A “DNA crunching” linear motor mechanism that employs a grip-and-release transient spring like compression of B- to A-form DNA has been found in our previous studies. Our FRET measurements in vitro show a decrease in distance from TerL to portal during packaging; furthermore, there is a decrease in distance between closely positioned dye pairs in the Y-stem of translocating Y-DNA that conforms to B- and A- structure. In normal translocation into the prohead the TerL motor expels all B-form tightly binding YOYO-1 dye that cannot bind A-form. The TerL motor cannot package A-form dsRNA. Our work reported here shows that addition of helper B form DNA:DNA (D:D) 20mers allows increased packaging of heteroduplex A-form DNA:RNA 20mers (D:R), evidence for a B- to A-form spring motor pushing duplex nucleic acid. A-form DNA:RNA 25mers, 30mers, and 35mers alone are efficiently packaged into proheads by the TerL motor showing that a proposed hypothetical dehydration motor mechanism operating on duplex substrates does not provide the packaging motor force. Taken together with our previous studies showing TerL motor protein motion toward the portal during DNA packaging, our present studies of short D:D and D:R duplex nucleic acid substrates strongly supports our previous evidence that the protein motor pushes rather than pulls or dehydrates duplex substrates to provide the translocation into prohead packaging force.

## 1. Introduction

Viral nucleic acid packaging by ATP-driven motor proteins is a critical area of viral assembly research because of its bearing on DNA dynamics and structure, its relationship to condensed genome structure, and its potential in gene therapy [1,2]. Packaging of dsDNA into a DNA empty procapsid or prohead occurs by a highly conserved mechanism among large tailed duplex DNA containing bacteriophages and herpes viruses, although only phage proheads can be filled in vitro to form an infectious virion. The encapsidated DNA is comparably condensed to ~500 mg/mL in these virions, but only in the bacteriophages is the relationship of DNA packaging and ejection into a host cell related to knowledge of how the two ends of the linear mature DNA are formed and delivered. In phage T4, the first mature genomic DNA end packaged is also the first end delivered into the host cell [3]. In phages, a two subunit terminase packaging enzyme consumes large amounts of ATP (~2 bp/ATP) to drive long genomic DNAs through a phage portal entry and exit channel. DNA exits from the phage capsid only after attachment of a host cell binding long narrow tubular tail through which it passes along with ejection or E proteins packaged into the prohead before the DNA.

**Figure 1:**
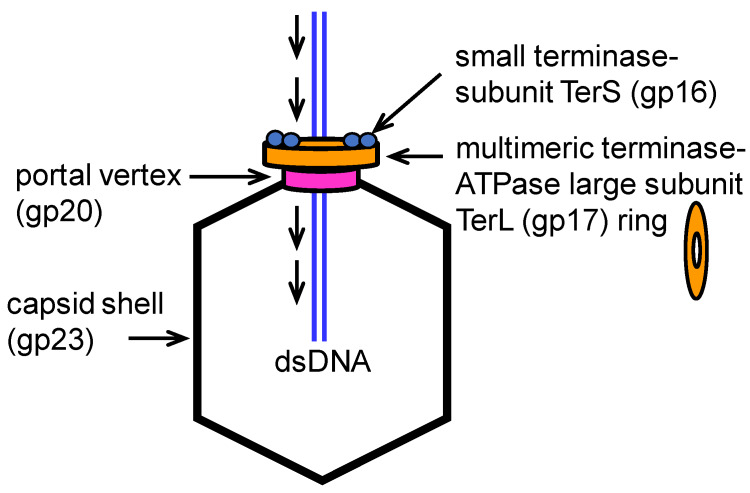
Conserved bacteriophage two subunit terminase prohead packaging; large terminase-ATPase subunit TerL (T4 gp17) only packages linear DNAs in vitro; small terminase subunit TerS (T4 gp16) required in vivo for concatemer (or circular DNA) packaging.

The large terminase subunit (TerL) drives packaging and cuts the long virtually endless head to tail linked genomic concatemer to form the mature genomic DNA (see Figure 1). TerL docked on the portal measures in conjunction with the portal a full genome’s worth or headful of DNA and then the TerL nuclease cuts it. The small terminase subunit (TerS) is involved in phage T4 packaging only in engaging the TerL nuclease activity to initiate cutting of the concatemer to make a packaging initiation end in the concatemer. In phage T4, TerL alone is able to package short 20 bp DNAs with ~100% efficiency. The small T4 terminase subunit is unnecessary and in fact inhibitory for packaging linear DNAs, although it is required for packaging circular or concatemeric DNA. Phage ATP-driven DNA translocation motors consist of a multimeric packaging terminase docked onto a unique prohead vertex containing a dodecameric portal ring. DNA is translocated into the empty prohead through the portal ring channel by a high force motor [4,5,6]. Even the Φ6 bacteriophage that packages three ssRNA segments, and then converts them to dsRNAs within the prohead, displays features analogous to dsDNA packaging, i.e. ATP-powered nucleic acid translocation through a channel containing a portal-like multimer that may function mechanistically like a helicase [7]. However, this motor likely operates by a different mechanism as it shows a lower translocation rate (20–60X less than the DNA rate), and our understanding of the RNA structure as related to motor dynamics is limited [1].

Among the tailed dsDNA phages with comparable packaging mechanisms, phage T4 has several advantages; e.g. diffusible proteins within the prohead; and packaging of any short (≤ 20 bp) DNA sequence by TerL alone into the prohead [8]. The phage Φ29, λ, and T4 motors all appear to produce comparable high (~60 pN (piconewtons)) force and rates sufficient to fill their proheads with a genome’s worth of DNA during development [9,10]. These TerL motors also all dock on the prohead portal at the C-terminal nuclease domain and at least in phage T4 the first end to enter the prohead is also the first end to leave the prohead through the attached prohead tail tube [3,11]. High resolution structures of all the packaging proteins (portal, large, and small terminase subunits) are now known in several phages [12,13,14,15,16]. 

In this paper, we report that the motor cannot package A-form dsRNA. Our work shows that addition of helper B form DNA:DNA (D:D) allows packaging of short heteroduplex A-form DNA:RNA (D:R), additional evidence for a B- to A-form spring motor. Interestingly, 25mer or longer D:R heteroduplexes are packaged with efficiencies comparable to the D:D mer of the same length.

## 2. Materials and Methods

### 2.1. Acq gp17 Purification

*Acq* gp 17 genes were cloned as the wt gp 17 gene in pTYB2 plasmids and were expressed in BL21 (DE3) pLysS. *Ac (residue 96 D) and q (residue 249 V)* gp17 was purified based on the Impact system of cloning (New England Biolabs, Ipswich, MA, USA) essentially as previously described [17]. The acridine and quinacrine residues that confer resistance to these two related intercalating dyes produce synergistic strong resistance of over ~10^7^ fold over wild type amino acid sequences TerL to these two intercalating dyes. Briefly, *acq* gp17 expressing cells were harvested and minced by sonication in Q buffer (50 mM Tris-HCl, pH 8.0, 100 mM NaCl, 0.1 mM EDTA, 5 mM MgCl_2_, 0.2 mM ZnCl_2_, and 1 mM ATP containing 10% glycerol). Then, the extract was spun at 15,000 rpm for 20 min, and the supernatant was removed and loaded onto a chitin column pre-equilibrated in Q buffer. The column was washed with 1–2 volumes of Q buffer, 2–3 column volumes of 1M NaCl and 0.5% Triton X-100 in Q buffer, and 1 column volume of Q buffer. In addition, 1 column volume of Q buffer with 40 mm dithiothreitol (DTT) was added, and the column was incubated overnight at 4 °C prior to eluting in Q buffer with 40 mM DTT. The eluted protein was dialyzed overnight against a Q buffer and then a Q buffer containing 30% glycerol to concentrate it. The purified TerL protein was aliquoted and stored at −80 °C.

### 2.2. Proheads Purification

Phage empty large proheads were purified by glycerol gradient centrifugation and column chromatography following infection with *16*amN66 – *17*amA465 – *13*amE111 – *rIIA*(ΔH88) as previously described [18]. The mutant infection accumulates proheads because of terminase L and S deficiency (*16* TerSam – *17TerL*am), which lack the prohead neck (*13* am) to prevent premature prohead-tail joining, and the proheads extract results from the *rIIA* (ΔH88) mutation to allow assay of phage formation by T4 wild-type DNA addition to the packaging mixture. The concentrated and partially purified proheads were loaded on a 15–45% glycerol gradient prepared in buffer A (50 mM Tris-HCl, pH 8.0, 5 mM MgCl_2_, 0.5 mM EDTA). Following centrifugation for 2 h at 30,000 rpm in a Beckman SW 50.1 rotor (Beckman, Indianapolis, IN, USA) at 4 °C, a relatively sharp prohead band that was visualized-three-fourths of the distance to the bottom of the tube was removed by side puncture. The glycerol gradient-purified proheads were then chromatographed by a linear gradient of NaCl to 0.5 M in buffer A on an FPLC DEAE column, which resolved two discrete peaks: empty large proheads (elps) and empty small proheads (esps). The proheads were concentrated by high speed centrifugation (18,000 rpm in a Sorvall SS34 rotor) and resuspended in buffer A.

### 2.3. Packaging Nuclease Assay

Packaging was carried out in 1× reaction buffer (50 mM Tris–HCl, pH 7.5, 6 mM MgCl_2_, 100 mM NaCl, 1.5 mM spermidine, 0.1 mg/mL bovine serum albumin, 1.5 mM DTT, 1.5 mM ATP, 5% polyethylene glycol (20k) (Fluka, Buchs, Switzerland) by mixing 50 nM large terminase (monomer), 10–20 nM DNA or DNA:RNA substrate, and 1–10 nM purified proheads in 16-μL volumes. Reaction tubes were incubated 1 h at room temperature: 1 μL of a 5-mg/mL DNase I and 10-mg/mL RNases (for DNA:RNA substrate) were then added, and tubes were incubated a further 30 min at 37 °C. A 1:1:1 mix of 0.5 M EDTA, 5 mg/mL proteinase K, and 10% SDS (3 μL) was added, and tubes were incubated at 65 °C for 30 min. Reactions were resolved on 10% (37.5:1) polyacrylamide gels developed in TBE buffer.

### 2.4. Fluorescence Correlation Spectroscopy (FCS) Measurements

Furthermore, 30-mer oligo was labeled with Cy5 (IDT, Coralville, IA) for FCS experiments. FCS measurements were performed in a confocal microscope (ISS Q2) with single-molecule detection sensitivity. The excitation source was a Fianium SC-400 super-continuum laser (NKT Photonics, Southampton, UK). An NKT super-select AOTF filter was used to select the excitation wavelength of 635 nm which was reflected by a dichroic mirror to a high-numerical-aperture (NA) water objective (60×; NA 1.2, Olympus, Japan) and focused onto the solution sample. The fluorescence was collected by avalanche photodiodes through a dichroic beam splitter and a band-pass (650–720nm; Chroma, Burlington, VT, USA) filter, thus eliminating the scattered excitation light and collecting the fluorescence from the Cy5 probes in the region of interest. The data acquisition was enabled by a B&H SPC-150 card operated in a photon time-tag time-resolved (TTTR) mode. ISS VistaVision software (ISS, Champaign, IL, USA) was used to analyze the FCS data to assess the in vitro binding of 30mer-heteroduplex to prohead. We determined the translational diffusion coefficients of 30mer D:R and the corresponding complexes with prohead. The percentage of fluorescent D:R that shifts into the more slowly diffusing species which is the prohead-bound fraction reflects the relative magnitude of packing efficiency. The FCS measurements and analyses were performed similarly to previous reports [11,19].

## 3. Results

**Figure 2:**
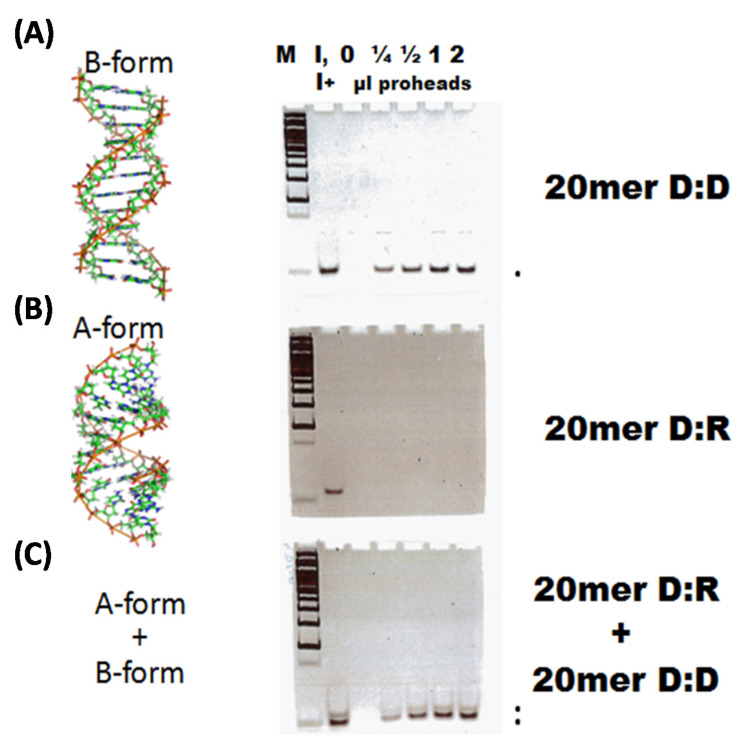
Nuclease assay showing packaging of (**A**) 20mer D:D, (**B**) 20mer D:R, and (**C**) 20mer D:R together with 20mer D:D substrates. Addition of a 20mer dsDNA helper (D:D) packaging substrate allows efficient packaging of a 20mer DNA:RNA heteroduplex (D:R), whereas the D:R alone is inefficient packaging. The D and R oligonucleotide complementary sequences are the same. Structures of B-form DNA (helix rise per base pair is ~3.4 Å) and A-form DNA (helix rise per base pair is ~2.6 Å) are shown. dsRNA and D:R heteroduplex structures are known to be A–form. M is Mol Wt marker lane, I is input packaging substrate, with µl of proheads added to the in vitro packaging mix shown above the indicated lanes. Following nuclease digestion as described under Materials and Methods, the amount of packaging-protected ds oligonucleotide stained with EthBr is shown for each substrate or substrate mixture as indicated by the dots (.).

An in vitro packaging experiment with the 20mer D:R heteroduplex without helper B-form DNA displays packaging with poor efficiency. In contrast, nuclease assay using 20mer B-form DNA shows that efficient packaging of 20mer A-form DNA:RNA occurs when 20mer B-form DNA is also present. As seen in Figure 2, bottom gel, lanes 4–7 (Panel C) show the presence of both the packaged A-form and B-form within proheads. Thus, B-form DNA has a required helper effect on the D:R heteroduplex and shows that the motor can act to translocate the D:R heteroduplex efficiently with the aid of the D:D 20mer. This result strongly suggests that the motor is pushing these short 20mer ds oligonucleotides rather than pulling them into the prohead, since pulling would require ds oligonucleotides at least as long as the TerL translocation channel (~100 Å) [14] rather than 68 Å long ds oligonucleotides and pulling would not be expected to show a helper effect. The D:D 20mer is pushing the D:R 20mer (inefficiently packaged when by itself) through the motor and portal channels. This work provides strong support for a B-form to A-form spring-like packaging motor that is able to recognize the A-form D:R oligonucleotide but is not able to translocate it efficiently without the aid of the B-form D:D helper. 

DNA compression from B- to A-form, although less studied and understood than DNA melting or stretching, has been measured to be within the force generation of the T4 and Φ29 packaging motors of up to 70–80 pN (piconewtons), as measured by Bustamante and co-workers [10]. As we referenced in our initial DNA crunching paper [20] direct B-form to A-form-like compression measurement (magnetic force probe for nanoscale biomolecules [21]) estimates a force of ~4 pN as the compression force of a B- to A-form short DNA duplex. In “the scrunchworm hypothesis [22]” supporting our B-form to A-form packaging motor, Steve Harvey provides a thermodynamics-based calculation from work [23] using trifluoroethanol (TFE) for conversion of B-form to A-form; on this basis, he calculates the B to A force is about 50 pN. TFE is a very harsh and non-physiological treatment as Harvey agrees (personal communication). It is uncertain which compression of B-form to A-form value is most relevant to packaging in vivo, but either of the two measured values, differing by about an order of magnitude, supports the conclusion that a DNA B- to A-form motor is consistent with optical tweezers measurements of packaging motor forces that can actually be generated. 

**Figure 3:**
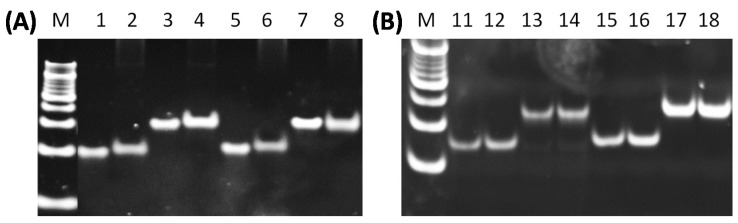
25, 30, and 35bp DNA:RNA each alone packaged efficiently. (**A**) 1, 2: input and packaging of 20D:D; 3, 4: input and packaging of 30D:D; 5, 6: input and packaging of 20D:R; 7, 8: input and packaging of 30D:R; (**B**) 11, 12: input and packaging of 25D:D; 13,14: input and packaging of 35D:D; 15,16: input and packaging of 25D:R; 17, 18: input and packaging of 35D:R. Note that nucleic acid in input samples is half of packaging samples.

Efficient packaging of D:R substrates of 25, 30, and 35 nucleotides alone without helper D:D duplexes was observed by nuclease assay (Figure 3). This shows that A-form D:R duplex nucleic acids of sufficient length can be translocated by the TerL motor without a helper D:D molecule push and does not require B-form to A-form dehydration. These longer D:R duplexes are packaged with efficiencies comparable to D:D duplexes with the same sequences. With larger amounts of D:Rs and longer packaging times, short 20mer D:Rs packaging is low without the aid of a 20mer D:D helper. The 20mer D:D helper effect (Figure 2C) as well as the efficient packaging of isolated longer 25, 30, and 35 D:R duplexes in the absence of a helper suggests that the TerL motor translocates by pushing on the 5’ D strand in D:R duplexes as previously speculated could be the case for the Phi6 packaging motor [24].

**Figure 4:**
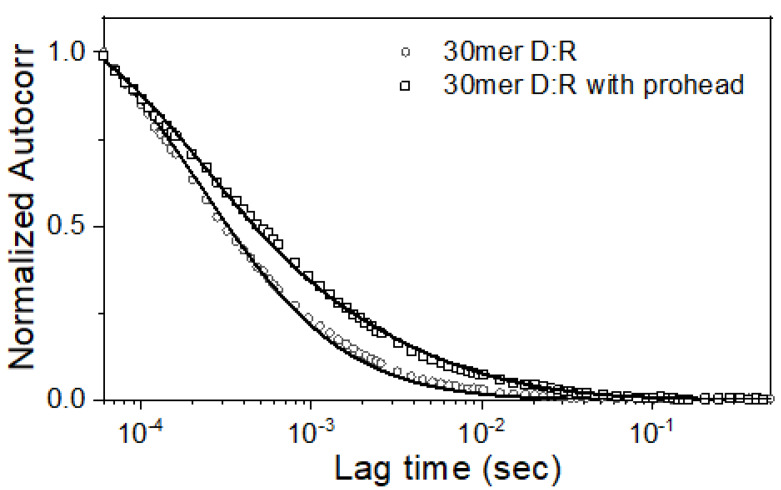
Autocorrelation plots of fluorescently labeled 30 mer D:R construct in the presence and absence of prohead. Experimental data are shown as points, and curves fitted with a one (30mer D:R) or two (30 mer D:R plus prohead) species diffusion model are superimposed as lines.

FCS is an excellent single molecule-based method that can determine the diffusion coefficients of the fluorescent species by determining the correlations between stochastic fluctuations in a sub-femtolitre reaction volume. Previously, we developed in vitro DNA packaging assay based on FCS determining the diffusion coefficients of short fluorescent DNA substrates as well as the slower diffusion coefficient of fluorescent DNA encapsidated into the much larger, slower moving proheads [3,8,11,17,19,20,25,26]. In this study, packaging reactions were set up using Cy5 tagged 30mer fluorescent oligos, unlabeled oligos and proheads, and analyzed by FCS after 60 min. The auto-correlation of the 30mer D:R substrate (i.e. one lacking proheads) could be fitted with a single species diffusion model implying a diffusion coefficient of around 100 μm^2^/s as demonstrated in Figure 4. By contrast, the correlation curves of the reactions in the presence of prohead was fitted by a two species diffusion model indicating the existence of two fluorescent species (Figure 4). The fit to the autocorrelation plot yielded a slower diffusion coefficient of 5 μm^2^/s with a relative abundance of 20%. The diffusion coefficient of the faster species of 100 μm^2^/s thus corresponded to 30mer free D:R substrate, while the slower species had a diffusion coefficient of T4 prohead. The FCS analyses thus suggest that approximately 20% of the input 30mer D:R substrate is sequestered in the prohead after a 60 min packaging reaction. We have observed the similar packaging efficiency (~20%) of the 30 mer D:D fluorescent substrate in the prohead as determined by an FCS assay. Overall, this concordance strongly implied that the both 30mer D:D or 30mer D:R substrates package with similar efficiency to the T4 prohead. 

## 4. Discussion

We previously reported that multiple short duplex DNAs can be packaged into T4 proheads by TerL alone in an ATP dependent translocation. TerS is unnecessary for translocation and in fact TerS inhibits short duplex DNA packaging. Multiple copies of short fluorescent dsDNAs have been demonstrated to be packaged into a single prohead by FCS-FRET. Associated multimers of TerL lead to the high ATPase-high DNA translocation form of TerL found on the prohead portal. TerS only promotes the initial TeL multimerization that is then retained by the TerL multimer bound on the prohead portal. There is no significant difference in packaging efficiency between very small (20 to 30 bp) and less small (40 to 100 bp) DNAs. However, <15 bp duplex base pair DNAs are not packaged, possibly as being too small for the translocation chamber of TerL. The fact that 20 or 30 bp duplex DNAs can be packaged however suggests that the substrate is pushed by the ~100 Å long packaging motor [14] rather than pulled by the motor. Importantly, 20 bp DNA:RNA heteroduplexes can be packaged more efficiently into T4 phage proheads by gp17 TerL in vitro with a 20 bp duplex D:D DNA helper (Figure 2). These findings show that, among very short duplex nucleic acids, one molecule acts as a translocation helper for another one by pushing it. Since it was previously demonstrated that long duplex RNAs are not packaged by the T4 TerL motor, likely the TerL motor must engage with the D strand to engage and translocate the duplex [8]. The current study is consistent with work on the Phi29 packaging motor that has been interpreted to suggest engagement with the 5’ strand [24].

A conformational change in the DNA is found not only in stalled Y-DNA, but accompanies active translocation of linear DNA into the prohead. Terminase gp17 packaging is inhibited in vivo and in vitro by a number of intercalating dyes (IDs) such as 9-aminoacridine, ethidium bromide, and YOYO-1 (a DNA high affinity dimeric ethidium bromide-like derivative). By use of FCS, nuclease, and Typhoon image assays, we find packaging strips all detectable YOYO-1 from the DNA when it enters the prohead in vitro, whereas YOYO-1 is still bound to the fast diffusing unpackaged substrate as measured by FCS. Comparably bulky covalently attached base dyes on duplex DNA (densely attached using dye modifiable 5’aminohexylacrylamido-dCTP:dCTP incorporated at a 2:1 ratio in RT synthesis of dsDNAs [2], and other dyes attached at the 5’-ends [3]) are successfully translocated by the terminase into the prohead. Thus, removal of all IDs from the substrate upon packaging strongly suggests that a transient conformational change in base pair stacking of the duplex accompanies linear DNA translocation, a novel finding for a DNA translocase that, unlike a helicase, is not expected to separate the two strands and thereby necessarily displace IDs. In support of this proposed A-form conformational change, whereas YOYO-1 binds to B-form, it does not bind to A-form DNA as shown by NMR [17].

**Figure 5:**
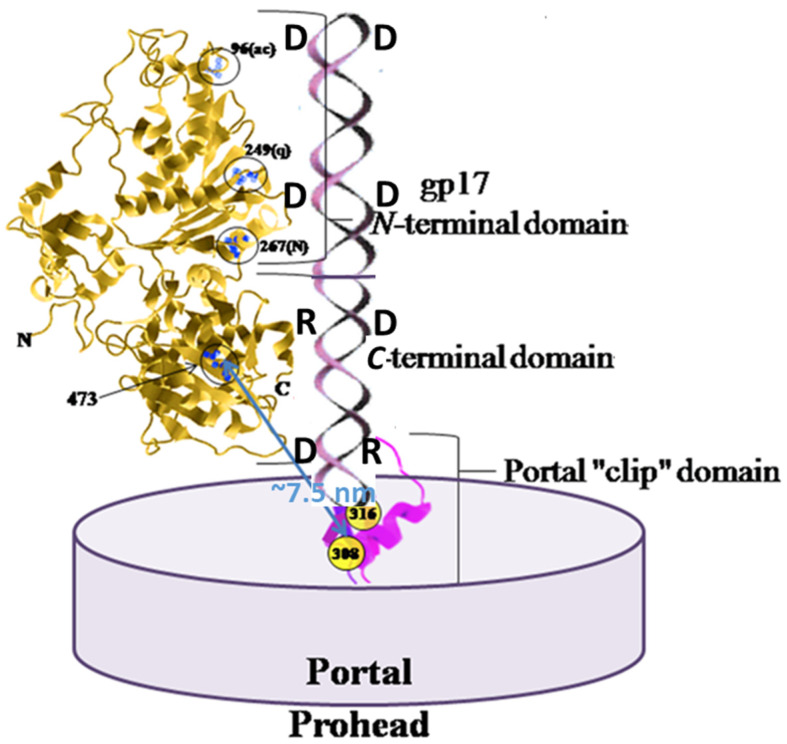
Phage T4 prohead portal gp20 binding to TerL from the C-terminal TerL nuclease containing domain. Two duplex nucleic acid segments are depicted as being translocated by the TerL ATP-driven motor; where the top segment is pushing the bottom segment through the portal tunnel into the prohead. TerL intercalating ac and q dye resistance mutations at residues 96, 249 are circled; mutations at residue 267 are closely coordinated with mutations at the ac and q sites; the circled residue 473 *ts* DNA translocation mutation is suppressed by portal *cs* mutation clip domain residue 308 (blue arrow). A FRET determined distance of ca.7.5 nm between portal clip domain Alexa488 maleimide dye labeled residue 316 and the terminase C-terminal ReAsH dye is found in this study (blue arrow). Structures are not to scale, nor are the numbers of terminase or portal monomers represented in the multimeric structure constituting the two-component packaging motor complex.

In summary, the present work provides strong support for a B-form to A-form spring-like packaging motor that is able to recognize and package A-form D:R oligonucleotides. Overall, this study supports a “DNA crunching” linear motor mechanism by the large TerL motor protein. This mechanism involves a grip-and-release compression of B- to A-form-like DNA by the N-terminal ATPase domain of the large subunit gripping the DNA as a consequence of TerL pushing upon the translocating DNA. Whether this is a consequence of a push upon a segment of the DNA that is resisted by a catch segment of the DNA (see Figure 5) or whether it results from a push of the duplex against a catch within the portal remains to be established. The packaging of short 20mer D:Rs is inefficient without the aid of a 20mer D:D helper to push the short D:R duplex. Importantly, however, 25-, 30-, and 35- mer D:Rs are packaged with efficiencies comparable to the D:Dmers of the same length. Since duplex D:R polynucleotides in solution are established to be in A-form, this work is inconsistent with the hypothesis that the TerL motor operates by a coupled DNA dehydration translocation mechanism dependent upon B-form to A-form transformation of DNA or of DNA:RNA heteroduplexes. A coupled dehydration translocation motor force mechanism has been proposed several times [22,27,28] and has been argued to be consistent with a biophysical theory [29,30] as well observations of changes in isolated duplex DNA conformation in solution as well as observed in association with prohead portals in solution. However, this proposed mechanism has thus far lacked direct experimental support, whereas our experimental observations reported here strongly conflict with a proposed dehydration motor force mechanism.
